# Poly[(μ_2_-4,4′-bipyridine)(μ_2_-3,5-dicarboxybenzenesulfonato)silver(I)]

**DOI:** 10.1107/S160053681000406X

**Published:** 2010-02-06

**Authors:** Dan Lin, Ping Lian, Yong-Rong Xie

**Affiliations:** a Department of Adult Education, Xinyu College, Xinyu, Jiangxi 338000, People’s Republic of China; b College of Chemistry and Life Science, Gannan Normal University, Ganzhou, Jiangxi 341000, People’s Republic of China

## Abstract

In the title compound, [Ag(C_8_H_5_O_7_S)(C_10_H_8_N_2_)]_*n*_, the Ag atom is tetra­coordinated by two 4,4′-bipydidine (4,4′-bipy) N atoms and two monodentate sulfonate O atoms of the 5-sulfoisophthalic acid (H_3_ *sip*) ligands. Adjacent Ag atoms are bonded through four sulfonate O atoms, forming a dinuclear unit with an Ag⋯Ag separation of 3.384 (5) Å; they are further linked together by the 4,4′-bipy lignds into a chain. Classical inter­molecular O—H⋯O and non-classical intra­molecular C—H⋯O hydrogen bonds are also observed in the two-dimensional supra­molecuar structure.

## Related literature

For general background to the design and construction of coordination polymers using multifunctional ligands, see: James (2003 ▶); Kawando & Fujita (2007 ▶); Liu *et al.* (2007 ▶, 2008 ▶). For related structures, see: Liu & Xu (2005 ▶, 2006 ▶); Lu *et al.* (2007 ▶). For the synthesis of related compounds, see: Wang *et al.* (2008 ▶). 
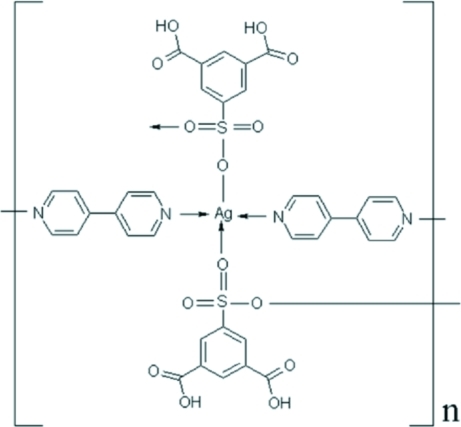


## Experimental

### 

#### Crystal data

[Ag(C_8_H_5_O_7_S)(C_10_H_8_N_2_)]
*M* *_r_* = 509.24
Triclinic, 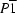

*a* = 7.9424 (16) Å
*b* = 9.970 (2) Å
*c* = 11.650 (2) Å
α = 83.38 (3)°
β = 87.36 (3)°
γ = 78.67 (3)°
*V* = 898.3 (3) Å^3^
*Z* = 2
Mo *K*α radiation
μ = 1.29 mm^−1^
*T* = 295 K
0.52 × 0.34 × 0.13 mm

#### Data collection

Siemens SMART CCD area-detector diffractometer
Absorption correction: multi-scan (*SADABS*; Sheldrick, 1996 ▶) *T*_min_ = 0.615, *T*_max_ = 0.831
7152 measured reflections
3156 independent reflections
2350 reflections with *I* > 2σ(*I*)
*R*_int_ = 0.030

#### Refinement

*R*[*F* ^2^ > 2σ(*F* ^2^)] = 0.044
*wR*(*F* ^2^) = 0.189
*S* = 1.06
3156 reflections
263 parameters
H-atom parameters constrained
Δρ_max_ = 0.97 e Å^−3^
Δρ_min_ = −1.43 e Å^−3^

### 

Data collection: *SMART* (Siemens, 1994 ▶); cell refinement: *SAINT* (Siemens, 1994 ▶); data reduction: *SAINT*; program(s) used to solve structure: *SHELXS97* (Sheldrick, 2008 ▶); program(s) used to refine structure: *SHELXL97* (Sheldrick, 2008 ▶); molecular graphics: *SHELXTL* (Sheldrick, 2008 ▶); software used to prepare material for publication: *SHELXTL*.

## Supplementary Material

Crystal structure: contains datablocks I, global. DOI: 10.1107/S160053681000406X/rk2187sup1.cif

Structure factors: contains datablocks I. DOI: 10.1107/S160053681000406X/rk2187Isup2.hkl

Additional supplementary materials:  crystallographic information; 3D view; checkCIF report

## Figures and Tables

**Table 1:** Hydrogen-bond geometry (Å, °)

*D*—H⋯*A*	*D*—H	H⋯*A*	*D*⋯*A*	*D*—H⋯*A*
O4—H4*B*⋯O5^i^	0.82	1.81	2.627 (8)	173
O6—H6*B*⋯O2^ii^	0.82	1.86	2.630 (8)	155
C1—H1*A*⋯O1^iii^	0.93	2.56	3.299 (10)	136
C6—H6*A*⋯O1^iv^	0.93	2.49	3.212 (9)	135
C12—H12*A*⋯O3	0.93	2.59	2.926 (9)	102
C16—H16*A*⋯O6	0.93	2.39	2.703 (10)	100
C16—H16*A*⋯O6^ii^	0.93	2.48	3.376 (9)	162

Symmetry codes: (i) 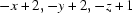; (ii) 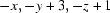; (iii) 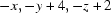; (iv) 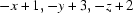.

**Fig. 1.**
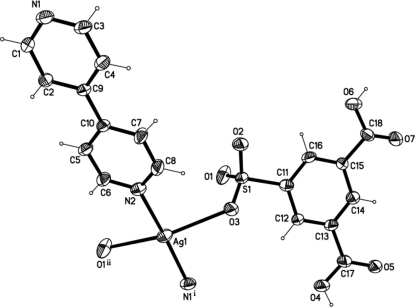
View of the structure of title compound, showing the atom-labelling scheme. Displacement ellipsoids are drawn at the 35% probability level. H atoms are shown as small spheres of arbitrary radius.

**Fig. 2.**
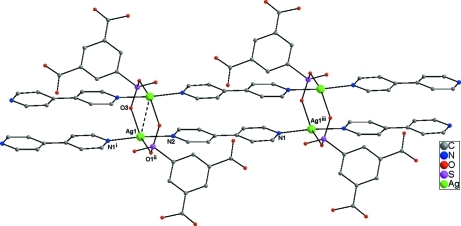
View of the 1-D chain in the crystal structure of title compound.

## supplementary crystallographic information

### Comment

The design and construction of coordination polymers from multifunctional ligands with metal ions is one of the most active areas of materials research. The inceasing interest in these materials is stimulated by their intriguing structural diversities and potential applications such as catalysis, molecular magnets and adsorption (James, 2003; Kawando *et al.*, 2007; Liu *et al.*, 2007 and 2008).

As a multidentate *O*-donor organic aromatic polycarboxylate ligand, 5-sulfoisophthalic acid monosodium salt (NaH_2_*sip*) has been used a good organic ligand 'spacer' to obtain many metal-organic complexes (Liu & Xu, 2005 and 2006; Lu *et al.*, 2007; Wang *et al.*, 2008). In this work, with the introduction of a rigid linear ligand, 4,4'-bipy, a novel 1-*D* organic-inorganic hybrid, Ag(H_2_*sip*)(bipy)(I) (H_3_*sip* = 5-sulfoisophthalic acid and bipy = 4,4'-bipydidine), has been obtained through hydrothermal self-assembly.

As depicted in Fig. 1, each asymmetric unit in compound I contains one Ag ion, one H_2_*sip* and one 4,4'-bipy ligands. The Ag1 ion is tetra-coordinated by two 4,4'-bipy nitrogen atoms with Ag—N distances being 2.194 (39) and 2.184 (40)Å, and two monodentate sulfonate oxygen atom with Ag—O bond lengths of 2.653 (27) and 2.621 (9)Å. The adjacent two Ag atoms are bonded through two oxygen atoms (O1 and O3) from one sulfonate group and its symmtery-related atoms to form a dinuclear unit with the Ag1···Ag1 seperation of 3.384 (5)Å. Such dinuclear units are further linked together by the linkage of 4,4'-bipy to construct 1-*D* chain-like, as shown in Fig. 2.

In compound I,each 4,4'-bipy ligand bridges two Ag1 ions to yield a 1-*D* chain along the *a* axis. While each H_2_*sip*^-^ ligand acts as mono-armed ligand using its bidentate sulfonate group, with remaining two carboxylate group uncoordinated.

In the crystal structure of the compound I, classical inter-molecular O—H···O and non-classical intra-molecular C—H···O hydrogen bonds are observed, which link the H_2_*sip*^-^and 4,4'-bipy molecules into a two-dimensional H-bonded network and stabilize the crystal packing.

### Experimental

AgNO_3_ (0.086 g, 0.50 mmol) and NaH_2_*sip* (0.133 g, 0.50 mmol) were dissolved in 10 mL of distilled water by vigorous stirring. 4,4'-bipy (0.078 g, 0.50 mmol) was added to the mixture and stirred for 30 min. The resulting solution was sealed in a teflon-lined stainless autoclave and heated at 433 K for 4 days under autogenous pressure and then cooled to room temperature during 18 h. Yellow block crystals of I suitable for X-ray analysis were collected in 59% yield (based on silver).

### Refinement

All H atoms bound to carbon were refined using a riding model with C—H = 0.93Å and *U*_iso_(H) = 1.2*U*_eq_(C). Two hydroxy H atoms were located in a difference map and refined with O—H distance restraints of 0.82Å and with *U*_iso_(H) = 1.5*U*_eq_(O).

### Crystal data

**Table:**

[Ag(C_8_H_5_O_7_S)(C_10_H_8_N_2_)]	*Z* = 2
*M**_r_* = 509.24	*F*(000) = 508
Triclinic, *P*1	*D*_x_ = 1.883 Mg m^−^^3^
Hall symbol: -P 1	Mo *K*α radiation, λ = 0.71073 Å
*a* = 7.9424 (16) Å	Cell parameters from 7152 reflections
*b* = 9.970 (2) Å	θ = 3.0–25.0°
*c* = 11.650 (2) Å	µ = 1.29 mm^−^^1^
α = 83.38 (3)°	*T* = 295 K
β = 87.36 (3)°	Block, yellow
γ = 78.67 (3)°	0.52 × 0.34 × 0.13 mm
*V* = 898.3 (3) Å^3^	

### Data collection

**Table:**

Siemens SMART CCD area-detector diffractometer	3156 independent reflections
Radiation source: fine-focus sealed tube	2350 reflections with *I* > 2σ(*I*)
graphite	*R*_int_ = 0.030
ω scans	θ_max_ = 25.0°, θ_min_ = 3.0°
Absorption correction: multi-scan (*SADABS*; Sheldrick, 1996)	*h* = −9→9
*T*_min_ = 0.615, *T*_max_ = 0.831	*k* = −11→11
7152 measured reflections	*l* = −13→13

### Refinement

**Table:**

Refinement on *F*^2^	Secondary atom site location: difference Fourier map
Least-squares matrix: full	Hydrogen site location: inferred from neighbouring sites
*R*[*F*^2^ > 2σ(*F*^2^)] = 0.044	H-atom parameters constrained
*wR*(*F*^2^) = 0.189	*w* = 1/[σ^2^(*F*_o_^2^) + (0.0986*P*)^2^ + 4.7322*P*] where *P* = (*F*_o_^2^ + 2*F*_c_^2^)/3
*S* = 1.06	(Δ/σ)_max_ < 0.001
3156 reflections	Δρ_max_ = 0.97 e Å^−^^3^
263 parameters	Δρ_min_ = −1.43 e Å^−^^3^
0 restraints	Extinction correction: *SHELXS97* (Sheldrick, 2008), Fc^*^=kFc[1+0.001xFc^2^λ^3^/sin(2θ)]^-1/4^
Primary atom site location: structure-invariant direct methods	Extinction coefficient: 0.009 (2)

### Special details

**Table:**

Geometry. Alls.u.'s (except the s.u. in the dihedral angle between two l.s. planes) are estimated using the full covariance matrix. The cell s.u.'s are taken into account individually in the estimation of s.u.'s in distances, angles and torsion angles; correlations between s.u.'s in cell parameters are only used when they are defined by crystal symmetry. An approximate (isotropic) treatment of cell s.u.'s is used for estimating s.u.'s involving l.s. planes.
Refinement. Refinement of *F*^2^ against ALL reflections. The weighted *R*-factor *wR* and goodness of fit *S* are based on *F*^2^, conventional *R*-factors *R* are based on *F*, with *F* set to zero for negative *F*^2^. The threshold expression of *F*^2^ > σ(*F*^2^) is used only for calculating *R*-factors(gt) *etc*. and is not relevant to the choice of reflections for refinement. *R*-factors based on *F*^2^ are statistically about twice as large as those based on *F*, and *R*-factors based on ALL data will be even larger.

### Fractional atomic coordinates and isotropic or equivalent isotropic displacement parameters (Å^2^)

**Table:**

	*x*	*y*	*z*	*U*_iso_*/*U*_eq_	
Ag1	0.65733 (8)	1.58243 (6)	0.94443 (6)	0.0538 (3)	
S1	0.3909 (2)	1.50090 (19)	0.72770 (16)	0.0418 (5)	
N1	−0.1357 (8)	2.4038 (6)	0.9399 (6)	0.0446 (15)	
N2	0.4706 (8)	1.7746 (6)	0.9457 (6)	0.0467 (15)	
C1	−0.0690 (10)	2.3342 (8)	1.0376 (7)	0.050 (2)	
H1A	−0.1067	2.3686	1.1072	0.060*	
C2	0.0521 (10)	2.2150 (7)	1.0411 (7)	0.0442 (18)	
H2A	0.0957	2.1723	1.1116	0.053*	
C3	−0.0763 (10)	2.3507 (8)	0.8420 (7)	0.0492 (19)	
H3A	−0.1186	2.3969	0.7722	0.059*	
C4	0.0425 (10)	2.2327 (8)	0.8396 (7)	0.0433 (17)	
H4A	0.0794	2.2015	0.7688	0.052*	
C5	0.2986 (9)	1.9505 (8)	1.0438 (7)	0.0432 (17)	
H5A	0.2617	1.9832	1.1141	0.052*	
C6	0.4124 (10)	1.8288 (7)	1.0428 (7)	0.0423 (17)	
H6A	0.4513	1.7813	1.1131	0.051*	
C7	0.2962 (11)	1.9698 (8)	0.8416 (7)	0.051 (2)	
H7A	0.2599	2.0159	0.7704	0.061*	
C8	0.4094 (12)	1.8445 (8)	0.8457 (7)	0.054 (2)	
H8A	0.4445	1.8074	0.7769	0.065*	
C9	0.1099 (9)	2.1579 (7)	0.9401 (6)	0.0366 (15)	
C10	0.2368 (8)	2.0268 (7)	0.9412 (6)	0.0343 (15)	
C11	0.4218 (9)	1.3809 (7)	0.6227 (6)	0.0406 (16)	
C12	0.5797 (9)	1.2933 (7)	0.6153 (6)	0.0409 (17)	
H12A	0.6681	1.2990	0.6630	0.049*	
C13	0.6045 (9)	1.1976 (8)	0.5365 (6)	0.0415 (17)	
C14	0.4729 (10)	1.1858 (8)	0.4675 (7)	0.0443 (17)	
H14A	0.4894	1.1182	0.4171	0.053*	
C15	0.3146 (9)	1.2759 (8)	0.4736 (6)	0.0420 (17)	
C16	0.2894 (9)	1.3741 (8)	0.5512 (6)	0.0446 (18)	
H16A	0.1847	1.4348	0.5553	0.054*	
C17	0.7761 (10)	1.1076 (8)	0.5217 (7)	0.0471 (19)	
C18	0.1776 (10)	1.2684 (8)	0.3903 (7)	0.0461 (18)	
O1	0.3287 (9)	1.4280 (6)	0.8299 (5)	0.0643 (17)	
O2	0.2644 (7)	1.6189 (5)	0.6839 (5)	0.0522 (14)	
O3	0.5564 (7)	1.5339 (7)	0.7414 (6)	0.0626 (17)	
O4	0.8908 (7)	1.1182 (7)	0.5940 (6)	0.071 (2)	
H4B	0.9825	1.0691	0.5782	0.106*	
O5	0.8050 (7)	1.0323 (6)	0.4427 (5)	0.0567 (15)	
O6	0.0381 (8)	1.3629 (8)	0.4059 (7)	0.083 (2)	
H6B	−0.0409	1.3479	0.3695	0.125*	
O7	0.1923 (8)	1.1889 (7)	0.3209 (6)	0.0678 (18)	

### Atomic displacement parameters (Å^2^)

**Table:**

	*U*^11^	*U*^22^	*U*^33^	*U*^12^	*U*^13^	*U*^23^
Ag1	0.0480 (4)	0.0396 (4)	0.0663 (5)	0.0174 (3)	−0.0110 (3)	−0.0155 (3)
S1	0.0382 (10)	0.0395 (10)	0.0434 (10)	0.0099 (8)	−0.0101 (8)	−0.0122 (8)
N1	0.033 (3)	0.039 (3)	0.058 (4)	0.002 (3)	−0.004 (3)	−0.005 (3)
N2	0.041 (3)	0.040 (3)	0.054 (4)	0.008 (3)	−0.007 (3)	−0.009 (3)
C1	0.046 (4)	0.048 (4)	0.054 (5)	0.008 (4)	−0.010 (4)	−0.025 (4)
C2	0.049 (4)	0.028 (3)	0.053 (4)	0.007 (3)	−0.017 (4)	−0.009 (3)
C3	0.041 (4)	0.048 (4)	0.049 (5)	0.013 (4)	−0.006 (3)	−0.004 (4)
C4	0.041 (4)	0.043 (4)	0.042 (4)	0.004 (3)	0.002 (3)	−0.009 (3)
C5	0.038 (4)	0.044 (4)	0.042 (4)	0.009 (3)	−0.001 (3)	−0.009 (3)
C6	0.045 (4)	0.035 (4)	0.042 (4)	0.006 (3)	−0.004 (3)	−0.008 (3)
C7	0.067 (5)	0.038 (4)	0.037 (4)	0.019 (4)	−0.009 (4)	−0.011 (3)
C8	0.064 (5)	0.046 (4)	0.049 (5)	0.014 (4)	−0.012 (4)	−0.023 (4)
C9	0.031 (3)	0.032 (3)	0.044 (4)	0.005 (3)	−0.006 (3)	−0.009 (3)
C10	0.030 (3)	0.029 (3)	0.043 (4)	0.000 (3)	−0.004 (3)	−0.009 (3)
C11	0.037 (4)	0.038 (4)	0.042 (4)	0.003 (3)	−0.007 (3)	0.000 (3)
C12	0.034 (4)	0.044 (4)	0.042 (4)	0.008 (3)	−0.008 (3)	−0.015 (3)
C13	0.032 (4)	0.045 (4)	0.041 (4)	0.007 (3)	−0.003 (3)	−0.003 (3)
C14	0.043 (4)	0.040 (4)	0.048 (4)	0.003 (3)	−0.006 (3)	−0.010 (3)
C15	0.038 (4)	0.047 (4)	0.038 (4)	0.005 (3)	−0.009 (3)	−0.013 (3)
C16	0.032 (4)	0.051 (4)	0.043 (4)	0.011 (3)	0.000 (3)	−0.002 (3)
C17	0.040 (4)	0.045 (4)	0.051 (4)	0.008 (3)	−0.012 (3)	−0.008 (4)
C18	0.043 (4)	0.045 (4)	0.050 (4)	0.000 (3)	−0.017 (3)	−0.012 (4)
O1	0.090 (5)	0.049 (3)	0.046 (3)	0.005 (3)	0.011 (3)	−0.009 (3)
O2	0.049 (3)	0.042 (3)	0.061 (3)	0.011 (2)	−0.017 (3)	−0.010 (3)
O3	0.044 (3)	0.070 (4)	0.077 (4)	0.006 (3)	−0.019 (3)	−0.041 (3)
O4	0.039 (3)	0.083 (5)	0.087 (5)	0.021 (3)	−0.019 (3)	−0.047 (4)
O5	0.044 (3)	0.060 (3)	0.064 (4)	0.012 (3)	−0.014 (3)	−0.030 (3)
O6	0.048 (4)	0.101 (5)	0.099 (5)	0.027 (4)	−0.031 (4)	−0.056 (4)
O7	0.059 (4)	0.075 (4)	0.068 (4)	0.011 (3)	−0.023 (3)	−0.038 (4)

### Geometric parameters (Å, °)

**Table:**

Ag1—N1^i^	2.178 (6)	C6—H6A	0.9300
Ag1—N2	2.181 (6)	C7—C10	1.374 (10)
Ag1—O3	2.658 (7)	C7—C8	1.385 (10)
Ag1—O1^ii^	2.626 (6)	C7—H7A	0.9300
S1—O3	1.436 (6)	C8—H8A	0.9300
S1—O1	1.441 (6)	C9—C10	1.485 (9)
S1—O2	1.449 (5)	C11—C12	1.386 (10)
S1—C11	1.784 (8)	C11—C16	1.387 (10)
N1—C1	1.336 (10)	C12—C13	1.379 (10)
N1—C3	1.340 (10)	C12—H12A	0.9300
N1—Ag1^iii^	2.178 (6)	C13—C14	1.379 (10)
N2—C6	1.334 (10)	C13—C17	1.492 (10)
N2—C8	1.346 (10)	C14—C15	1.399 (10)
C1—C2	1.372 (10)	C14—H14A	0.9300
C1—H1A	0.9300	C15—C16	1.389 (10)
C2—C9	1.385 (10)	C15—C18	1.508 (10)
C2—H2A	0.9300	C16—H16A	0.9300
C3—C4	1.358 (10)	C17—O5	1.239 (9)
C3—H3A	0.9300	C17—O4	1.295 (9)
C4—C9	1.383 (10)	C18—O7	1.183 (9)
C4—H4A	0.9300	C18—O6	1.326 (10)
C5—C6	1.364 (10)	O4—H4B	0.8200
C5—C10	1.396 (10)	O6—H6B	0.8200
C5—H5A	0.9300		
			
O3—Ag1—N2	92.9 (2)	C8—C7—H7A	119.5
O3—Ag1—N1^i^	89.0 (2)	N2—C8—C7	122.4 (7)
O3—Ag1—O1^ii^	158.2 (3)	N2—C8—H8A	118.8
N2—Ag1—N1^i^	174.0 (2)	C7—C8—H8A	118.8
N2—Ag1—O1^ii^	88.5 (2)	C4—C9—C2	115.1 (6)
O3—S1—O1	113.3 (4)	C4—C9—C10	123.1 (7)
O3—S1—O2	113.0 (4)	C2—C9—C10	121.8 (6)
O1—S1—O2	111.8 (4)	C7—C10—C5	115.5 (6)
O3—S1—C11	105.9 (3)	C7—C10—C9	122.3 (6)
O1—S1—C11	104.6 (4)	C5—C10—C9	122.1 (6)
O2—S1—C11	107.6 (3)	C12—C11—C16	120.8 (7)
C1—N1—C3	115.9 (6)	C12—C11—S1	118.6 (5)
C1—N1—Ag1^iii^	120.9 (5)	C16—C11—S1	120.6 (5)
C3—N1—Ag1^iii^	123.1 (5)	C13—C12—C11	119.4 (7)
C6—N2—C8	117.0 (6)	C13—C12—H12A	120.3
C6—N2—Ag1	122.8 (5)	C11—C12—H12A	120.3
C8—N2—Ag1	120.2 (5)	C14—C13—C12	120.8 (7)
N1—C1—C2	123.7 (7)	C14—C13—C17	118.6 (7)
N1—C1—H1A	118.2	C12—C13—C17	120.6 (7)
C2—C1—H1A	118.2	C13—C14—C15	119.7 (7)
C1—C2—C9	120.4 (7)	C13—C14—H14A	120.1
C1—C2—H2A	119.8	C15—C14—H14A	120.1
C9—C2—H2A	119.8	C16—C15—C14	119.8 (7)
N1—C3—C4	123.3 (7)	C16—C15—C18	121.5 (7)
N1—C3—H3A	118.4	C14—C15—C18	118.6 (7)
C4—C3—H3A	118.4	C11—C16—C15	119.4 (7)
C3—C4—C9	121.5 (7)	C11—C16—H16A	120.3
C3—C4—H4A	119.3	C15—C16—H16A	120.3
C9—C4—H4A	119.3	O5—C17—O4	123.6 (7)
C6—C5—C10	121.1 (7)	O5—C17—C13	121.1 (7)
C6—C5—H5A	119.5	O4—C17—C13	115.3 (7)
C10—C5—H5A	119.5	O7—C18—O6	124.6 (7)
N2—C6—C5	123.0 (7)	O7—C18—C15	124.7 (7)
N2—C6—H6A	118.5	O6—C18—C15	110.7 (6)
C5—C6—H6A	118.5	C17—O4—H4B	109.5
C10—C7—C8	120.9 (7)	C18—O6—H6B	109.5
C10—C7—H7A	119.5		

Symmetry codes: (i) *x*+1, *y*−1, *z*; (ii) −*x*+1, −*y*+3, −*z*+2; (iii) *x*−1, *y*+1, *z*.

### Hydrogen-bond geometry (Å, °)

**Table:**

*D*—H···*A*	*D*—H	H···*A*	*D*···*A*	*D*—H···*A*
O4—H4B···O5^iv^	0.82	1.81	2.627 (8)	173
O6—H6B···O2^v^	0.82	1.86	2.630 (8)	155
C1—H1A···O1^vi^	0.93	2.56	3.299 (10)	136
C6—H6A···O1^ii^	0.93	2.49	3.212 (9)	135
C12—H12A···O3	0.93	2.59	2.926 (9)	102
C16—H16A···O6	0.93	2.39	2.703 (10)	100
C16—H16A···O6^v^	0.93	2.48	3.376 (9)	162

Symmetry codes: (iv) −*x*+2, −*y*+2, −*z*+1; (v) −*x*, −*y*+3, −*z*+1; (vi) −*x*, −*y*+4, −*z*+2; (ii) −*x*+1, −*y*+3, −*z*+2.
